# Correlation of Coagulation Parameters With Clinical Outcomes During the Coronavirus-19 Surge in New York: Observational Cohort

**DOI:** 10.3389/fphys.2021.618929

**Published:** 2021-02-23

**Authors:** Morayma Reyes Gil, Jesus D. Gonzalez-Lugo, Shafia Rahman, Mohammad Barouqa, James Szymanski, Kenji Ikemura, Yungtai Lo, Henny H. Billett

**Affiliations:** ^1^Department of Pathology, Albert Einstein College of Medicine, Montefiore Medical Center, New York City, NY, United States; ^2^Division of Hematology, Department of Medical Oncology, Albert Einstein College of Medicine, Montefiore Medical Center, New York City, NY, United States; ^3^Department of Epidemiology and Population Health, Albert Einstein College of Medicine, Montefiore Medical Center, New York City, NY, United States; ^4^Division of Hematology, Departments of Oncology and Medicine, Albert Einstein College of Medicine, Montefiore Medical Center, New York City, NY, United States

**Keywords:** coagulopathy, D-Dimer, COVID-19, New York City, prothrombin time

## Abstract

**Importance:**

COVID-19 has caused a worldwide illness and New York became the epicenter of COVID-19 in the United States from Mid-March to May 2020.

**Objective:**

To investigate the coagulopathic presentation of COVID and its natural course during the early stages of the COVID-19 surge in New York. To investigate whether hematologic and coagulation parameters can be used to assess illness severity and death.

**Design:**

Retrospective case study of positive COVID inpatients between March 20, 2020-March 31, 2020.

**Setting:**

Montefiore Health System main hospital, Moses, a large tertiary care center in the Bronx.

**Participants:**

Adult inpatients with positive COVID tests hospitalized at MHS.

**Exposure (for observational studies):**

Datasets of participants were queried for demographic (age, sex, socioeconomic status, and self-reported race and/or ethnicity), clinical and laboratory data.

**Main Outcome and Measures:**

Relationship and predictive value of measured parameters to mortality and illness severity.

**Results:**

Of the 225 in this case review, 75 died during hospitalization while 150 were discharged home. Only the admission PT, absolute neutrophil count (ANC) and first D-Dimer could significantly differentiate those who were discharged alive and those who died. Logistic regression analysis shows increased odds ratio for mortality by first D-Dimer within 48 hrs. of admission. The optimal cut-point for the initial D-Dimer to predict mortality was found to be 2.1 μg/mL. 15% of discharged patients required readmission and more than a third of readmitted patients died (5% of all initially discharged).

**Conclusion:**

We describe here a comprehensive assessment of hematologic and coagulation parameters in COVID-19 and examine the relationship of these to mortality. We demonstrate that both initial and maximum D-Dimer values are biomarkers that can be used for survival assessments. Furthermore, D-Dimer may be useful to follow up discharged patients.

## Background

COVID-19 is a heterogenous disease caused by 2019-nCoV/SARS-CoV-2 virus, a new member of the coronavirus family. Clinical manifestations vary from an asymptomatic illness in some to rapid death in others (Chen et al., 2020; Huang C. et al., 2020; Wu et al., 2020; Zhou et al., 2020). COVID-19 has caused a worldwide illness, causing the World Health Organization to declare this a pandemic on January 30th 2020. (Cucinotta and Vanelli, 2020) The United States is currently one of the global hot spots with a fatality rate ranging from 10% to 27% among adults aged more than 85 years, 3 to 11% among adults of age group 65-84 years and 1% to 3% among age group 55-64 years (Cucinotta and Vanelli, 2020). New York became the epicenter of COVID-19 in the United States and the Bronx had the highest prevalence, hospitalizations and deaths per capita in New York during mid-March to May 2020 (Goyal et al., 2020; NYC, 2020; Wadhera et al., 2020).

Our hospital is a large tertiary care center and is the primary medical system for the Bronx. Faced with a human crisis of enormous proportions, we decided to try and understand the potential correlations with illness severity and death and the natural course of both complicated and less complicated disease during the first surge in New York. Coagulopathy and D-Dimer elevations are reported in 3.75-68.0% of the COVID-19 patients (Berger et al., 2020; Gungor et al., 2020; Huang Y. et al., 2020; Valerio et al., 2020; Yao et al., 2020; Yu H. H. et al., 2020; Yu B. et al., 2020; Zhang L. et al., 2020). Initial studies from patients in Wuhan showed an association of high D-Dimer, a marker for thrombosis, with mortality (Tang et al., 2020). A recent study from Manhattan, New York, showed that 76% of COVID + patients that required hospitalization had elevated D-Dimer on admission and those patients were more likely to developed critical illness and complications including thrombotic events, kidney injury and death (Berger et al., 2020). The primary objective of this study is to examine baseline, and dynamic changes in D-Dimer and other laboratory data and to determine the relation between these laboratory parameters, in particular tests of coagulation, to illness severity.

Herein we studied if D-Dimer correlated with other laboratory parameters and clinical complications, including thrombosis and mortality in the Bronx population.

## Methods

### Study Design

#### Data Gathering and Variables

In our retrospective, single-center study, we included confirmed COVID-19 cases in Montefiore Medical Center/University Hospital for Albert Einstein College of Medicine, Moses Campus, who were hospitalized and had routine coagulation tests done between March 20th to March 31st 2020. For those samples without an ordered D-Dimer, D-Dimer was performed alongside with prothrombin time (PT) as part of this study. All cases were established with reverse-transcriptase–polymerase-chain-reaction real-time (RT PCR) assay of the nasal and the pharyngeal swabs. We excluded patients younger than 18 years of age, those admitted for “COVID-19-like” illnesses but negative initial test results, and patients for whom data were missing. The study was approved by the Albert Einstein College of Medicine Institutional Review Board. Electronic medical records of the patients were reviewed to obtain epidemiological, demographic, clinical and laboratory data.

These variables included demographic attributes (age, sex, and self-reported race and/or ethnicity) and baseline comorbidities (body mass index, previous history of hypertension, diabetes, kidney, pulmonary, liver, autoimmune, cancer, or sickle cell disease on presentation). Initial vital signs, including oxygen saturation, and laboratory values were documented. Obesity was defined as BMI more than 30. Cancer was defined as malignancy with active treatment or diagnosed within the last 5 years. Laboratory values consisted of a complete blood count, a metabolic profile, assessments of liver and renal function, procalcitonin and coagulation testing [prothrombin time (PT), partial thromboplastin time (PTT), D-Dimer values, fibrinogen]. Management and clinical outcomes were followed up to June 10th 2020. We assessed for interventions and time to interventions including ICU admission, intubation, thrombosis and anticoagulation, mortality, hospital discharge and post-discharge readmission. Thrombosis was document only if a thrombus was identified on radiological imaging. *Ex vivo* clotting while on hemodialysis (HD) or continuous renal replacement therapy (CRRT) was based on the need for kit/filter change and/or visual clots as documented in the clinical progress notes. We documented anticoagulation medications given to each patient within 48 h preceding the thrombus or clotting event. Doses for prophylactic anticoagulation were: apixaban 2.5 mg twice a day, enoxaparin 40 mg subcutaneously once a day (BMI < 40, GFR = 30) or enoxaparin 30 mg subcutaneously twice a day (BMI = 40). Therapeutic anticoagulation doses were apixaban 5 mg twice a day, enoxaparin 1-5 mg/kg/day (1 mg/kg/day if GFR = 30) or 1 mg/kg twice a day. All intravenous unfractionated heparin (UFH) administrations were deemed therapeutic, typically 80 units/kg IV bolus followed by continuous infusion of 18 units/kg/h or 5000 units IV bolus followed by continuous infusion of 1300 units/h. All intravenous bivalirudin administrations were deemed therapeutic. Patients on warfarin prior to admission that continued warfarin while hospitalized were considered on a therapeutic regimen.

The earliest symptoms were categorically defined: New onset cough, dyspnea, and diarrhea, intubation and dialysis requirements. Maximum and, when appropriate, minimum values were noted and the day of these values from admission date were noted. Levels of parameters of particular interest were recorded daily whenever possible. Chest imaging done on presentation to the emergency department was documented.

### Laboratory Testing

All cases were established with reverse-transcriptase–polymerase-chain-reaction real-time (RT PCR) assay of the nasal and the pharyngeal swabs. Coagulation tests (prothrombin time, D-Dimer, partial thromboplastin time and fibrinogen) were performed by STA-R Max instruments. STA Liatest LIA D-Dimer assay was performed as per manufacturer recommendations and reported as FEU μg/mL with a cut-off of <0.5 ug/ml to rule out PE. Complete blood counts were performed by Sysmex XN9000. Chemistry assays were performed by Roche instrumentation and reagents as per manufacturer recommendations.

### Statistical Methods

Data analysis was performed using R software, version 3.6.2. Differences in demographic, clinical variables and laboratory assessments between patients who died in the hospital and patients discharged alive were compared using chi-square tests, or Fisher’s exact tests for categorical variables and two-sample Student *t*-tests, or the Mann-Whitney *U*-test for continuous variables. Logistic regression was carried out to examine the relationship between the factors and lab parameters under examination and in-hospital mortality. Parameters for the logistic regression analysis were selected based on a *p*-value = 0.1 (Bursac et al., 2008).

The receiver operating characteristic curves (ROC), Youden’s J statistics and Kaplan-Meier were used to assess performance of D-Dimer in the first 48 h on predicting in-hospital mortality adjusted for age, O2 saturation and sex.

## Results

### Study Population: Admission and Mortality Data

Of the 225 patients who tested positive for COVID, 75 (33%) patients died during hospitalization while 150 (67%) were discharged alive. Analysis of demographics, comorbidities, and clinical parameters are included in Table 1. Similar to other reports, patients that succumbed to death were significantly older than survivors (median [interquartile range (IQR)]; 71.00 [62.0, 77.5] vs. 59.00 [48.3, 67.0] years, *p* < 0.001). Among pre-existing comorbidities, hypertension (74.7% vs. 60.0%, *p* = 0.03), diabetes (48.0% vs. 34.0%, *p* = 0.04) and cancer (12.0% vs. 4.6%, *p* = 0.04) were more prevalent in non-survivors. The only clinical parameter on admission that showed statistical significance between non-survivors vs. survivors were the oxygen saturation on RA (median [interquartile range (IQR)]; 90.00 [80.00, 95.00] vs. 96.00 [92.00, 97.00], *p* < 0.001). We could not detect a statistical significance for gender or race between survivors vs. non-survivors in this population.

**TABLE 1:** Demographics and clinical features of COVID positive patients at admission.

Demographics/Clinical Features	Died *n* = 75	Discharged *n* = 150	*P*-value
Age, yrs. (median (IQR))	71.0 (62.0, 77.5)	59.0 (48.3, 67.0)	<0.001
**Gender**			
Female, n (%)	26 (34.7)	69 (46.0)	0.139
Male, n (%)	49 (65.3)	81 (54.0)	
**Comorbidity**			
Hypertension, n (%)	56 (74.7)	90 (60.0)	0.03
Diabetes, n (%)	36 (48.0)	51 (34.0)	0.04
Chronic Kidney Disease, n (%)	17 (22.6)	26 (17.3)	0.92
Pulmonary Disease, n (%)	17 (22.6)	38(25.3)	0.19
Liver Disease, n (%)	4 (5.3)	6 (4.0)	0.21
Autoimmune Disease, n (%)	5 (6.7)	8 (5.5)	0.16
Cancer, n (%)	9 (12.0)	7 (4.6)	0.04
Sickle Cell Disease, n (%)	1 (1.3)	1 (0.7)	0.25
**Race**			
Black, n (%)	35 (46.7)	54 (36.0)	0.361
White, n (%)	11 (14.7)	29 (19.3)	
Asian, n (%)	2 (2.7)	2 (1.3)	
Other/Declined, n (%)	27 (36.0)	65 (43.3)	
**Ethnicity**			
Hispanic, n (%)	20 (26.7)	57 (38.0)	0.1
Non- Hispanic, n (%)	51 (68.0)	75 (50.0)	
Other/Declined, n (%)	4 (5.3)	18 (12.0)	
BMI (mean)	30.5 (8.28)	29.7 (6.08)	0.46
**Presentation**			
Fever, n (%)	55 (73.3)	104 (69.3)	0.641
Cough, n (%)	54 (72.0)	100 (66.7)	0.51
SOB, n (%)	56 (74.7)	98 (65.3)	0.205
Diarrhea, n (%)	9 (12.0)	33 (22.0)	0.102
Infiltrate on initial X-ray, n (%)	69 (92)	136 (90.6)	0.11
O2 Sat (median (IQR))	90.0 (80.0, 95.0)	96.0 (92.3, 97.0)	<0.001

### Clinical Characteristics

As shown in Table 2, intubation (*p* < 0.00001), cardiac arrest (*p* < 0.001), dialysis requirement (*p* < 0.001) and significant liver disease) (liver enzyme elevation greater than 2.5 fold the upper limit of normal range, *p* < 0.034) during hospitalization were all associated with decreased survival (*p* < 0.001). The overall length of stay for survivors discharged home was significantly shorter than for patients that died during hospitalization.

**TABLE 2:** Laboratory and clinical course of COVID positive patients during hospitalization.

	Died *n* = 75	Discharged *n* = 150	*P*
**Lab Test**			
**Absolute Neutrophilic Count, ×10^9^/L (median (IQR))**			
- Initial (median (IQR))	5.40 (3.45, 7.70)	4.45 (3.23, 6.60)	0.038
- Minimum (median (IQR))	4.70 (3.0, 6.75)	3.00 (2.20, 4.00)	<0.001
- Hospital days to minimum	1.00 (0.00, 3.00)	3.00 (1.00, 5.00)	<0.001
- Maximum (median (IQR))	12.60 (9.60, 16.50)	5.95 (4.20, 9.03)	<0.001
- Hospital days to maximum	7.00 (3.00, 11.00)	3.00 (0.00, 5.00)	<0.001
**Absolute Lymphocytic Count, ×10^9^/L (median (IQR))**			
- Initial (median (IQR))	0.90 (0.60, 1.20)	0.90 (0.70, 1.28)	0.457
- Minimum (median (IQR))	0.50 (0.40, 0.80)	0.80 (0.60, 1.10)	<0.001
- Hospital days to minimum	3.00 (1.50, 5.00)	1.00 (0.00, 4.00)	<0.001
**Hemoglobin, g/dl (median (IQR))**			
- First	12.70 (10.45, 14.25)	13.10 (11.72, 14.20)	0.48
- Minimum	10.00 (7.50, 11,60)	11.30 (9.30, 12.80)	0.001
- Hospital days to minimum	6.00 (3.00, 10.00)	4.00 (2.00, 7.00)	0.003
**Platelets, ×10^9^/L (median (IQR))**			
- Initial	193.00 (153.75, 236.25)	198.00 (145.50, 245.00)	0.793
- Minimum	150.00 (120.50, 193.50)	178.00 (136.00, 218.00)	0.004
- Hospital days to minimum	2.00 (0.00, 4.00)	1.00 (0.00, 2.00)	0.003
**Procalcitonin, ng/ml (median (IQR))**			
- Initial (median (IQR))	0.10 (0.10, 0.50)	0.30 (0.10, 1.40)	0.17
- Maximum (median (IQR))	2.00 (0.00, 8.25)	0.00 (0.00, 1.00)	0.0011
**Initial AST (median (IQR))**	51.00 (29.50, 78.50)	38.00 (27.00, 60.00)	0.29
**Initial ALT (median (IQR))**	29.00 (18.00, 41.50)	28.00 (19.00, 43.75)	1.0
**Initial Creatinine**	1.40 (1.10, 2.00)	1.00 (0.80, 1.37)	0.41
**Clinical Course**			
Cardiac arrest (%)	36 (48.0)	0 (0.0)	<0.001
Dialysis required (%)	39 (52.0)	15 (10.0)	<0.001
LFT = 2.5x ULN at any time	15 (20.0)	16 (10.7)	0.034
Intubation required (%)	47 (62.7)	17 (11.3)	<0.00001
Days to intubation (mean)	1.00 (0.00, 3.00)	1.00 (0.00, 4.00)	0.68
Intubated days (mean)	6.00 (3.00, 11.00)	9.00 (5.00, 11.75)	0.292
LOS, days (median (IQR))	10.00 (6.00, 14.50)	7.00 (4.00, 12.00)	0.012

### Laboratory Data

Table 2 contains non-coagulation labs and Table 3 contains coagulation labs. The only significantly different admission lab tests between deceased vs. survivors were absolute neutrophil count PT and D-Dimer within first 48 h. (Tables 2, 3). D-Dimer results within first 48 h. of admission were missing in a significant number of patients. The lack of D-Dimer data however did not translate into any statistical differences in other values, such as demographics, clinical and other laboratory characteristics. The same differences were observed among survivors and non-survivors in patients who were discharged who had D-Dimer values within 48 hrs. as were observed in those who did not (Tables 4, 5).

**TABLE 3:** Coagulation parameters and anticoagulation treatments during hospitalization.

	Died *n* = 75	Discharged *N* = 150	*p*
**Coagulation Profile**			
**PT** sec (median [IQR])			
- Initial	14.50 (13.70, 16.10)	13.80 (13.30, 14.65)	0.002
- Maximum (mean)	16.40 (15.20, 20.20)	14.70 (13.80, 16.30)	<0.001
- Hospital days to maximum (mean (SD))	4.00 (0.00, 9.00)	2.00 (0.00, 6.00)	0.273
**PTT** sec (Median (IQR))			
- Initial	34.00 (30.67, 38.15)	32.60 (29.65, 36.50)	0.109
- Maximum	41.20 (33.40, 50.30)	34.70 (31.20, 40.08)	<0.001
- Hospital days to maximum (mean (SD))	3.00 (0.00, 8.00)	1.00 (0.00, 2.00)	0.003
**D-Dimer** ug/ml FEU			
- Within first 48 h (Median (IQR))	2.85 (1.36, 10.36)	1.05 (0.64, 2.23)	<0.001
- Maximum (Median (IQR))	4.66 (2.16, 12.41)	1.19 (0.68, 3.01)	<0.0001
- Hospital days to maximum	5.00 (2.00, 10.00)	3.00 (1.00, 5.00)	0.006
**Fibrinogen** mg/dl (mean (SD))			
- Initial	641.00 (449.00, 736.00)	656.50 (505.25, 743.75)	0.64
- Maximum	690.91 (255.21)	715.00 (274.60)	0.75
- Hospital days to maximum (mean (SD))	5.00 (2.00, 11.50)	4.50 (2.00, 11.00)	0.839

**TABLE 4:** Characteristics of Discharged patients with initial D-Dimer < 48 h vs. > 48 h since admission.

Characteristics of Discharged Patients	1^*st*^ D-Dimer < 48 h	1^*st*^ D-Dimer > 48 h	*p*
n	96	54	
1st D-Dimer (median [IQR])	1.02 [0.64, 2.04]	1.18 [0.66, 3.18]	0.26
Age (mean (SD))	55.50 [46.75, 67.50]	60.00 [52.25, 66.00]	0.33
Sex = Male (%)	55 (57.3)	26 (48.1)	0.36
**Comorbidity**			
Hypertension, n (%)	53 (55.2)	37 (68.5)	0.16
Diabetes, n (%)	30 (31.2)	21 (38.9)	0.44
Chronic Kidney disease (%)	15 (14.9)	13 (22.8)	0.30
Pulmonary disease, n (%)	27 (26.7)	12 (21.1)	0.55
Liver Disease, n (%)	4 (4.2)	2 (3.7)	1.00
Autoimmune Disease, n (%)	4 (4.2)	4 (7.4)	0.64
Cancer, n (%)	5 (5.2)	2 (3.7)	1.00
Sickle Cell Disease, n (%)	0 (0.0)	1 (1.9)	0.77
**Race (%)**			
Black	32 (33.3)	22 (40.7)	0.27
White	16 (16.7)	13 (24.1)	
Asian	2 (2.1)	0 (0.0)	
Other	46 (47.9)	19 (35.2)	
**Ethnicity (%)**			
Declined	12 (12.5)	6 (11.1)	0.59
Hispanic	39 (40.6)	18 (33.3)	
Non-Hispanic	45 (46.9)	30 (55.6)	
**Presentation**			
Fever, n (%)	63 (65.6)	41 (75.9)	0.26
Cough, n (%)	67 (69.8)	33 (61.1)	0.37
SOB, n (%)	65 (67.7)	33 (61.1)	0.53
Diarrhea, n (%)	21 (21.9)	12 (22.2)	1
O2 Sat (median [IQR])	96.00 [91.75, 97.00]	96.00 [93.00, 97.00]	0.43
**Clinical laboratory data on admission**			
ANC×10^9^/L (median [IQR])	4.90 [3.60, 7.00]	4.05 [2.83, 5.56]	0.03
ALC×10^9^/L (median [IQR])	0.90 [0.68, 1.23]	0.90 [0.80, 1.28]	0.59
Hb g/dl (median [IQR])	13.10 [11.80, 14.20]	12.80 [11.55, 14.17]	0.58
PLT×10^9^/L (median [IQR])	202.50 [161.00, 240.25]	180.50 [145.00, 228.50]	0.12
PT (median [IQR])	13.80 [13.20, 14.40]	13.95 [13.40, 16.02]	0.16
PTT (median [IQR])	32.55 [30.03, 36.75]	32.60 [28.90, 35.25]	0.79
AST (median [IQR])	40.00 [28.00, 61.00]	33.00 [25.00, 54.00]	0.17
ALT (median [IQR])	28.00 [19.00, 48.00]	28.00 [18.00, 38.00]	0.32
CRT (median [IQR])	1.00 [0.80, 1.32]	1.00 [0.80, 1.37]	0.73
**Clinical Course**			
Cardiac arrest (%)	0 (0.0)	0 (0.0)	NA
Dialysis required (%)	8 (8.3)	7 (13.0)	0.54
LFTs = 2.5X ULN (%)	8 (8.3)	8 (14.8)	0.34

**TABLE 5:** Characteristics of patients that died during hospitalization with initial D-Dimer < 48 h vs. > 48 h since admission.

Characteristics of Deceased Patients	1^*st*^ D-Dimer < 48 h	1^*st*^ D-Dimer > 48 h	*p*
n	38	37	
1st D-Dimer (median [IQR])	1.85 [0.88, 3.16]	6.26 [2.25, 13.72]	0.003
Age (mean (SD))	73.00 [65.00, 78.00]	68.00 [56.00, 75.00]	0.13
Sex = Male (%)	25 (65.8)	24 (64.9)	1
**Comorbidity**			
Hypertension, n (%)	30 (78.9)	26 (70.3)	0.55
Diabetes, n (%)	20 (52.6)	16 (43.2)	0.56
Chronic Kidney disease (%)	5 (13.2)	12 (32.4)	0.09
Pulmonary disease, n (%)	7 (18.4)	10 (27.0)	0.54
Liver Disease, n (%)	2 (5.3)	2 (5.6)	1
Autoimmune Disease, n (%)	4 (10.5)	1 (2.7)	0.37
Cancer, n (%)	4 (10.5)	5 (13.5)	0.97
Sickle Cell Disease, n (%)	0 (0.0)	1 (2.7)	0.99
**Race (%)**			
Black	19 (50.0)	16 (43.2)	0.38
White	4 (10.5)	7 (18.9)	
Asian	2 (5.3)	0 (0.0)	
Other	13 (34.2)	14 (37.8)	
**Ethnicity (%)**			
Declined	0 (0.0)	4 (10.8)	0.09
Hispanic	12 (31.6)	8 (21.6)	
Non-Hispanic	26 (68.4)	25 (67.6)	
**Presentation**			
Fever, n (%)	27 (71.1)	28 (75.7)	0.85
Cough, n (%)	28 (73.7)	25 (69.4)	0.88
SOB, n (%)	28 (73.7)	28 (75.7)	1
Diarrhea, n (%)	6 (15.8)	3 (8.1)	0.50
O2 Sat (median [IQR])	86.00 [80.00, 94.75]	90.00 [80.00, 95.00]	0.67
**Clinical laboratory data on admission**			
ANC ×10^9^/L (median [IQR])	6.55 [4.23, 8.78]	5.10 [3.30, 6.00]	0.06
ALC ×10^9^/L (median [IQR])	0.85 [0.60, 1.20]	0.90 [0.80, 1.10]	0.49
Hb g/dl (median [IQR])	12.60 [10.70, 14.30]	12.70 [10.40, 14.20]	0.71
PLT ×10^9^/L (median [IQR])	194.50 [148.25, 241.50]	199.00 [144.00, 244.00]	0.90
PT (median [IQR])	14.60 [13.70, 16.10]	14.30 [13.60, 15.80]	0.60
PTT (median [IQR])	34.70 [30.52, 37.85]	33.30 [30.67, 38.23]	0.74
AST (median [IQR])	51.00 [32.00, 73.50]	51.00 [28.00, 85.00]	0.84
ALT (median [IQR])	25.00 [18.00, 40.75]	31.00 [20.00, 42.00]	0.34
CRT (median [IQR])	1.44 [1.12, 2.63]	1.30 [0.90, 1.80]	0.56
**Clinical Course**			
Cardiac arrest (%)	22 (57.9)	14 (37.8)	0.13
Dialysis required (%)	22 (57.9)	17 (45.9)	0.42
LFTs = 2.5X ULN (%)	6 (15.8)	9 (24.3)	0.53

**FIGURE 1:**
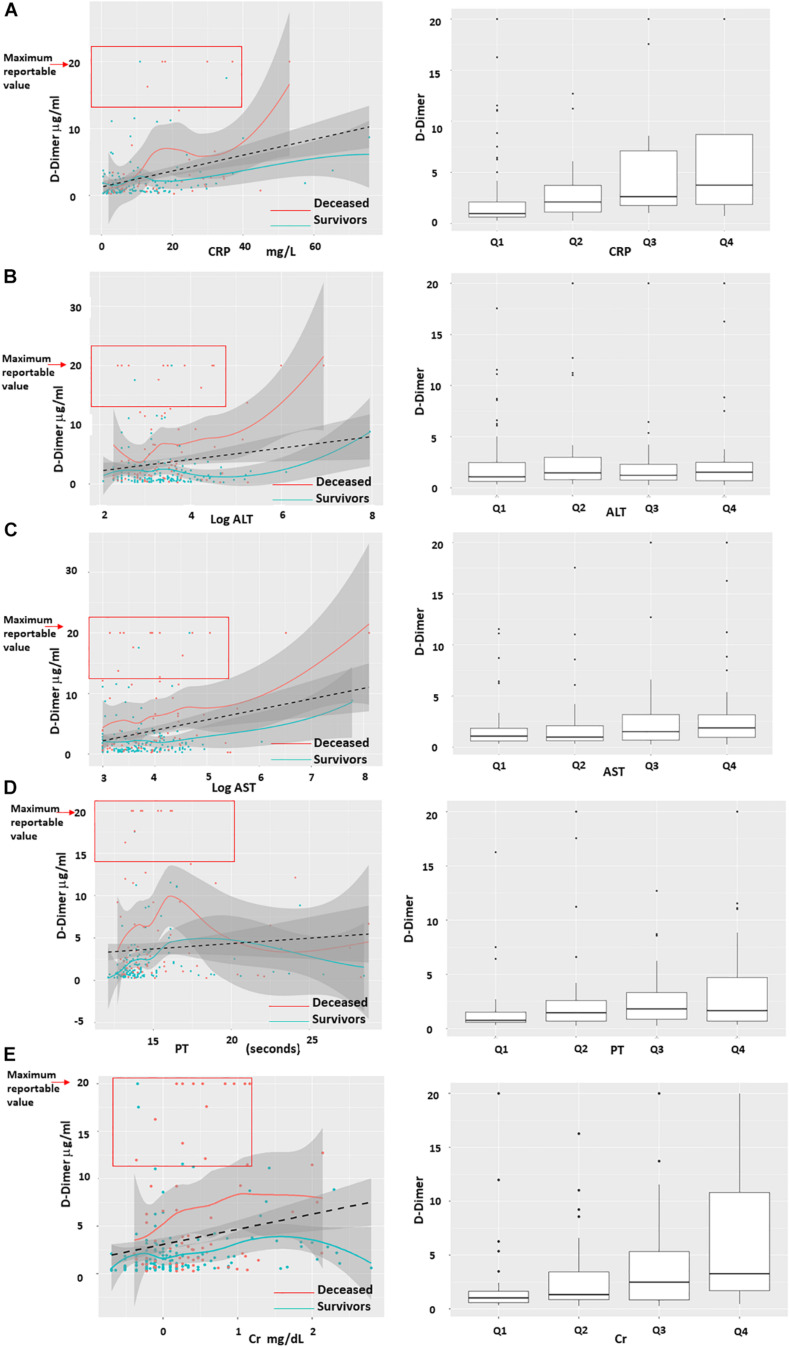
Regression plots of D-Dimer correlation with C-Reactive Protein, AST, ALT, PT and Creatinine. D-Dimer levels of patients that died (red) vs. patients that survived (blue) did not significantly correlate with C-Reactive Protein (CRP), AST, ALT, Prothrombin Time (PT) and Creatinine (Cr). **(A–E)** Left column shows density plots with 95% intervals (gray solid zones). Black dotted line = linear regression. Blue and red line = smooth regression, also known as “Distribution free” which assumes no correlation and finds the best fit to the trajectory of the points. Red square highlights that the majority of cases with maximum D-Dimer of >20 μg/mL were non-survivors and had corresponding levels of CRP, AST, ALT, PT, and Cr in the lowest quartiles. **(A–E)** Right columns represents the quartiles of CRP, AST, ALT, PT, and Cr distribution based on D-Dimer.

The maximum PT, PTT, D-Dimer, procalcitonin, absolute neutrophil count (ANC) were statistically significantly higher in non-survivors vs. survivors. Likewise, the minimum absolute lymphocyte count (ALC), hemoglobin and platelet count were statistically significantly lower in non-survivors compared to survivors. Inflammation can mildly increase D-Dimer. Likewise, renal and hepatic disease can increase D-Dimer levels due to decreased clearance. However, no obvious positive correlation was seen in regression plots of D-Dimer vs. CRP (inflammation marker), ALT and AST (liver function markers), PT and CRT (renal function marker) (Figures 1A-E).

On univariable logistic regression analysis, older age (OR = 1.06, 95%CI: 1.03–1.09; *p* < 0.001), hypertension (OR 3.04, 95%CI: 1.31-7.74, *p* = 0.01), diabetes (OR 2.44, 95%CI: 1.14-5.33, *p* = 0.02), lower oxygen saturation (OR 0.90, 95%CI: 0.85-0.94, *p* < 0.001), PT [OR = 1.17 (1.02-1.37, *p* = 0.028)] and increased first 48 h D-Dimer level (OR = 1.23, 95%CI 1.09–1.43; *p* = 0.003), were associated with increased odds for mortality. Using multivariable logistic analysis only D-Dimer, age and oxygen saturation remain statistically significant, and the odds ratio for D-Dimer increased slightly (OR = 1.24, 95%CI 1.04–1.49; *p* = 0.018), highlighting the strength of D-Dimer as an independent risk factor for mortality (Table 6).

**TABLE 6:** Logistic regression analysis of risk factors for mortality in hospitalized COVID patients.

Dependent: expired		no	yes	Odds ratio (univariable)	Odds ratio (multivariable)
D-Dimer < 48 h	Mean (SD)	1.7 (1.8)	4.4 (6.1)	1.23 (1.09-1.43, *p* = 0.003)	1.24 (1.04-1.49, *p* = 0.018)
Age	Mean (SD)	56.9 (16.9)	70.4 (12.6)	1.06 (1.03-1.09, *p* < 0.001)	1.06 (1.01-1.11, *p* = 0.011)
Sex	Female	41 (42.7)	13 (34.2)	-	-
	Male	55 (57.3)	25 (65.8)	1.43 (0.66-3.20, *p* = 0.367)	1.18 (0.32-4.35, *p* = 0.806)
Ethnicity	hispanic	39 (40.6)	12 (31.6)		
	not hispanic or declined	57 (59.4)	26 (68.4)	1.48 (0.68-3.37, *p* = 0.332)	1.18 (0.33-4.18, *p* = 0.801)
Diarrhea	no	75 (78.1)	32 (84.2)	-	-
	yes	21 (21.9)	6 (15.8)	0.67 (0.23-1.73, *p* = 0.431)	1.63 (0.35-7.62, *p* = 0.533)
HT	no	43 (44.8)	8 (21.1)	-	-
	yes	53 (55.2)	30 (78.9)	3.04 (1.31-7.74, *p* = 0.013)	1.93 (0.46-8.04, *p* = 0.365)
DM	no	66 (68.8)	18 (47.4)	-	-
	yes	30 (31.2)	20 (52.6)	2.44 (1.14-5.33, *p* = 0.023)	1.21 (0.34-4.32, *p* = 0.766)
Cancer	no	91 (94.8)	34 (89.5)	-	-
	yes	5 (5.2)	4 (10.5)	2.14 (0.50-8.56, *p* = 0.277)	1.05 (0.15-7.12 *p* = 0.961)
Oxygen saturation	Mean (SD)	93.6 (5.9)	83.7 (15.1)	0.90 (0.85-0.94, *p* < 0.001)	0.89 (0.83-0.95, *p* = 0.001)
ANC	Mean (SD)	6094.8 (4603.2)	7171.1 (3993.4)	1.00 (1.00-1.00, *p* = 0.214)	1.00 (1.00-1.00, *p* = 0.173)
PT	Mean (SD)	14.4 (2.4)	15.6 (3.2)	1.17 (1.02-1.37, *p* = 0.028)	1.10 (0.90-1.34, *p* = 0.366)
PTT	Mean (SD)	33.6 (5.6)	35.3 (6.8)	1.05 (0.98-1.13, *p* = 0.177)	1.04 (0.94-1.16, *p* = 0.437)
Chest infiltrates	no	9 (9.4)	3 (7.9)	-	-
	yes	87 (90.6)	35 (92.1)	1.21 (0.34-5.68, *p* = 0.787)	0.96 (0.13-7.32, *p* = 0.968)

**FIGURE 2:**
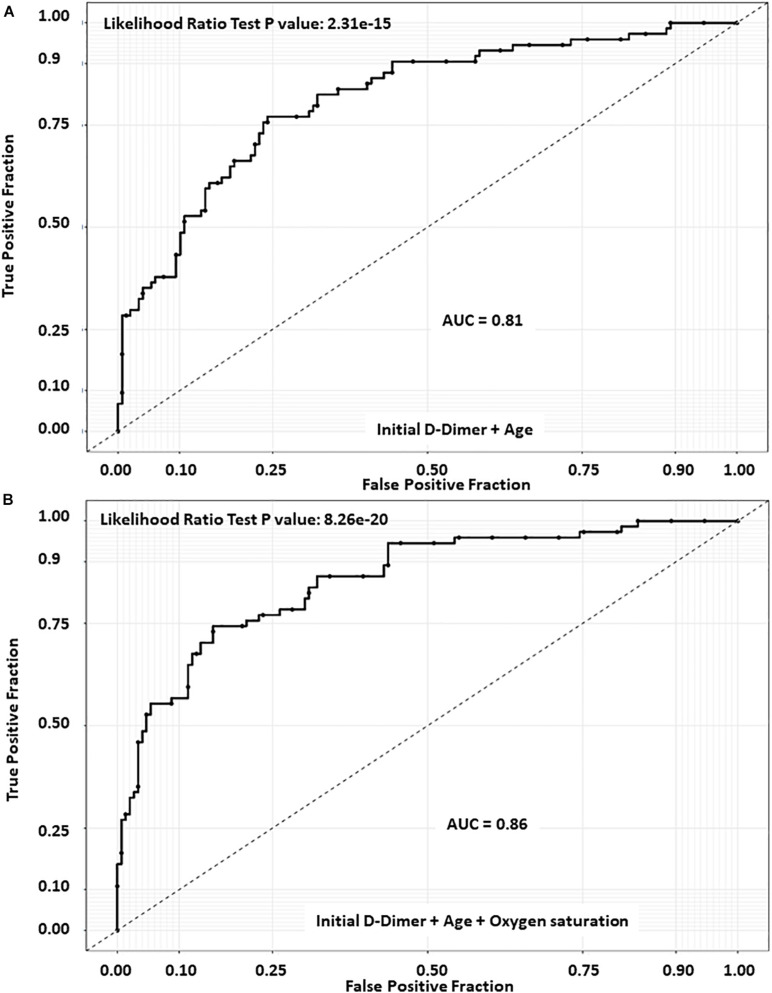
Receiver operating characteristic curve (ROC) of D-Dimer. **(A)** D-Dimer combined with age only displayed an AUC of 0.81. **(B)** D-Dimer combined with age and Oxygen saturation showed an area under the curve (AUC) of 0.86.

**FIGURE 3:**
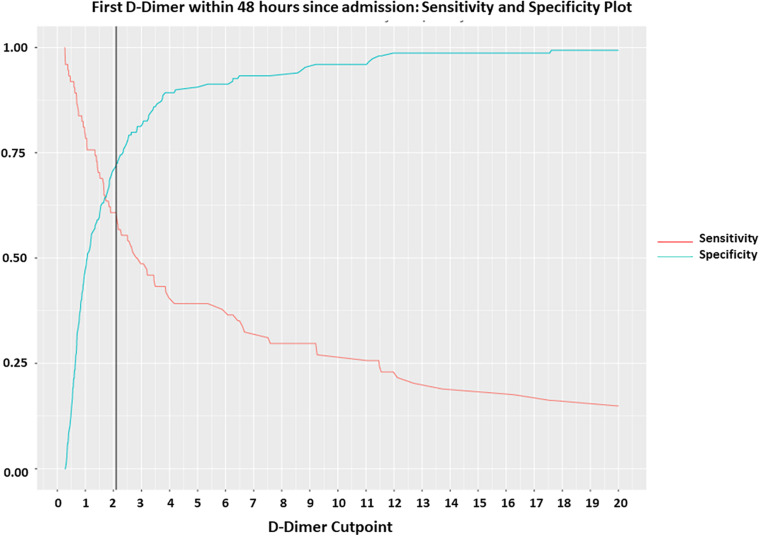
Optimal cut off of initial D-Dimer by Youden’s *J* statistics. Youden index measuring the optimal cut point for initial D-Dimer as a differentiating marker when equal weight is given to sensitivity and specificity for the values in the cohort. The optimal cut-point for the initial D-Dimer within 48 h since admission to predict mortality was found to be at 2.1 g/ml at a sensitivity of 0.61, specificity 0.73 and AUC 0.71.

**FIGURE 4:**
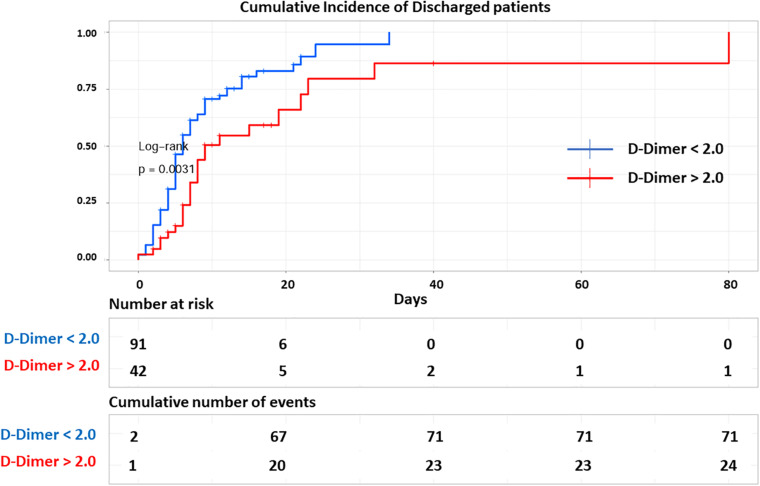
Cumulative discharged curve. Kaplan Meier curve shows cumulative number of discharged COVID-19 positive patients over time (*n* = 133) based on initial D-Dimer. Patients with initial D-Dimer < 2 ug/ml (blue) showed higher rate of discharge alive compared to patients with initial D-Dimer > 2 ug/ml (red) (log rank, *p* = 0.0031). Bottom table shows the number of COVID-19 positive patients admitted and at risk of mortality over time and the cumulative number of discharged patients in each group in increments of every 10 days. Each line represents a discharged patient.

The receiver operating characteristic curve (ROC) of first 48 h. D-Dimer adjusted for age and oxygen saturation, showed an area under the curve (AUC) of 0.86 and a very similar AUC 0.81 with only D-Dimer and age, underscoring D-Dimer as an important admission lab test as predictor of mortality (Figure 2). Using Youde’s J statistic, the optimal cut-point for the initial D-Dimer to predict mortality was found to be 2.1 μg/mL (Figure 3). The cumulative survival by Kaplan Meier using a cutoff of initial D-Dimer of 2 μg/mL shows a clear separation between the two groups: 78% (71/91) of patients with D-Dimer < 2 μg/mL survived whereas only 57% (24/42) of the patients with D-Dimer = 2 μg/mL survived (Figure 4).

### Thrombosis and Anticoagulation

A total of 10 patients (4.4%) had documented *in vivo* thrombosis, mainly venous thromboembolism (DVT). Although *in vivo* thrombosis was not significantly different between survivors and non-survivors, *ex vivo* clotting, mainly in hemodialysis lines, was significantly higher non-survivors compared to survivors. 187 of the 225 patients (83%) were on some anticoagulation. Of those who were on anticoagulation, 14.7% of the survivors were given therapeutic doses as compared to 21.3% of those who died. These data, and the medications involved, are detailed in Table 7. Maximum D-Dimer was not statistically significant between patients not anticoagulated compared to anticoagulated patients (Table 7). However, when comparing discharged vs. deceased patients, maximum D-Dimer was significantly higher in non-survivor patients not anticoagulated and prophylactically anticoagulated but not among patients that received therapeutic anticoagulation (Table 7). Among discharged patients maximum D-Dimer, but not initial D-Dimer, was significantly higher in patients that develop clots (including both *in vivo* and *ex vivo* clots). Whereas within patients that eventually died during hospitalization both initial D-Dimer and maximum D-Dimer levels were significantly higher in those that developed clots (Table 8).

**TABLE 7:** Thrombosis and anticoagulation in patients that died during hospitalization vs. patient discharged.

	Died (*n* = 75)	Discharged (*n* = 150)	*p*
Thrombosis	2 (2.7)	8 (5.3)	0.837
∙ Deep Venous Thrombosis	1 (1.3)	6 (4.0)	0.277
∙ Pulmonary Embolism	0 (0.0)	1 (0.7)	1
∙ Arterial Thrombosis	1 (1.3)	1 (0.7)	1
∙ Stroke	0 (0.0)	0 (0.0)	1
*Ex Vivo* Clotting	12 (16)	4 (2.7)	0.0002
**Anticoagulation, n (%)**			
None	14 (18.7)	24 (16.0)	0.902
Prophylactic	45 (60)	104 (69.3)	0.720
∙ Heparin	6 (8.0)	14 (9.3)	1.000
∙ Enoxaparin	38 (50.7)	89 (59.3)	0.899
∙ Apixaban	1 (1.3)	1 (0.7)	0.660
Therapeutic	16 (21.3)	22 (14.7)	0.576
∙ Heparin	5 (6.7)	5 (3.3)	1.000
∙ Enoxaparin	1 (1.3)	3 (2.0)	0.146
∙ Apixaban	5 (6.7)	12 (8.0)	0.718
∙ Bivalirudin	2 (2.7)	0 (0.0)	0.530
∙ Warfarin	3 (4.0)	2 (1.3)	1.000
**Maximum D-Dimer by anticoagulation**			
None, D-Dimer μg/ml FEU (Median (IQR))	7.58 (2.81, 11.02)	1.20 (0.70, 3.13)	0.03
Prophylactic, D-Dimer μg/ml FEU (Median (IQR))	3.45 (1.82, 17.07)	1.35 (0.68, 2.63)	<0.0001
Therapeutic, D-Dimer μg/ml FEU (Median (IQR))	6.18 (3.03, 11.16)	0.94 (0.71, 3.76)	0.11
One-Way ANOVA *p*-Value	0.29	0.99	

**TABLE 8:** Initial D-Dimer and maximum D-Dimer levels in discharged and expired patients that developed clots during hospitalization.

	Clot	No Clot	*P*-value
**Discharged patients**			
1st D-Dimer ug/ml FEU (Median (IQR))	1.64 (0.97, 5.50)	1.02 (0.63, 2.17)	0.20
Max D-Dimer ug/ml FEU (Median (IQR))	9.83 (4.33, 15.98)	1.13 (0.66, 2.61)	0.01
**Expired patients**			
1st D-Dimer ug/ml FEU (Median (IQR))	12.08 (5.49, 20)	2.16 (1.05, 6.6)	0.006
Max D-Dimer ug/ml FEU (Median (IQR))	14.48 (7.78, 20)	3.21 (1.7, 9.26)	0.002

### Sequela Post-discharge

In Table 9, 22 of the 150 (14.7%) patients that were discharged alive had complications that required readmission. Complications included respiratory (5/22), cardiovascular (4/22), renal (4/22), and infectious (9/22) etiologies. 8 patients (5% of the discharged patients) died due to post-discharge complications and all happened within 1 month since discharge.

**TABLE 9:** Description of complications in discharged patients that required readmission.

cases	Respiratory	Cardiovascular	Renal	Thrombosis	Infection	Readmission Days since discharge	D-Dimer ug/ml readmission	Death
66 M	Acute hypoxia RF				Septic shock	11	6.25	yes
38 F			AKI	Sickle cell VOC DVT, splenic infarct		26	ND	yes
72 M	Acute respiratory failure/ventilation				Aspiration PNA	27	1.20	
74 M		CAD				124	ND	
81 M					E Coli bacteremia	94	1.58	
50 M		Hypertensive urgency				71	ND	
75 M	Hypoxic RF					19	9.90	yes
62 F				PE/DVT		32	ND	
61 M		CHF				9	ND	yes
44 M					Acute cholecystitis	84	0.91	
80 M					epididymitis/ochitis	95	ND	
75 M	Hypoxic RF		AKI			6	2.44	yes
68 M					MRSA PNA	20	0.40	
51 M				RLE thrombosis		58	ND	
56 F			CKD HD			51	ND	
58 F			Volume overload/dialysis			6	ND	
35 M					Fever, viral URI	161	ND	
77 F				DVT	Extended spectrum beta-lactamase sepsis	7	2.31	yes
52 M		myocarditis				3	1.20	
81 F				R foot ischemia arterial occlusion		66	ND	
87 M	Hypoxic RF					14	13.5	yes
85 F					Septic shock	28	ND	yes

## Discussion

This study of COVID19 patients in the Bronx, NY, United States confirms the original observations made by Wuhan studies regarding the association of D-Dimer with mortality in COVID19 patients (Huang C. et al., 2020; Vidali et al., 2020). Goyal et al. (2020) described a population in New York City (NYC) that is majority White (37%), minority Black (12.5%) and unknown percentage of Hispanics, as no classification for Hispanics was provided. Richardson et al. (2020) also described the demographics and comorbidities of a NYC that is majority White (39.8%), followed by Hispanics (23%) and Blacks (22.6%). Although Richardson et al. (2020) showed laboratory data, no comparison or statistical analysis was shown between discharged patients vs. survivors. In contrast we studied a population of Blacks (40%), Hispanics (33%) and a minority Whites (18%), representative of the Bronx and overall NYC demographics and we were able to analyze physiological and laboratory parameters as predictors of mortality in our cohort of US COVID19 infected patients.

Our cohort consists of 225 patients seen at the main Montefiore Medical Center hospital at the beginning of the pandemic peak in NYC. Montefiore comprises a population of minorities that is largely underserved and understudied (Blacks and Hispanics with a minor population of Whites and Asians). Given the huge patient load, only the very sick were being admitted in the Bronx. Survival in our hospitalized patients during this period was poor, with 33% of the hospitalized patients dying. This is comparable to the death rate of 28.3% for the rest of New York City during the COVID-19 peak (Filardo et al., 2020; Kalyanaraman Marcello et al., 2020; Lamb et al., 2020; Thompson et al., 2020).

Although both male gender and Black race were increased in those who died vs. those who were discharged, we did not get a significant association. This is probably due to the smaller sample size in our cohort which limited comprehensive study of the influences of comorbidities, race and socioeconomic status. Indeed, larger studies have shown a relationship of Black race with increased mortality (Laurencin and McClinton, 2020), including a recent study from our same institution (Neugarten et al., 2020) that demonstrated that Blacks have a higher mortality even after adjusting by age and comorbidities.

Several published cohorts have examined laboratory characteristics of patient admissions (Huang C. et al., 2020; Richardson et al., 2020; Zhang L. et al., 2020). Concordantly with those, we show that PT and D-Dimer within first 48hrs of admission were associated with mortality, underscoring the association of COVID-19 coagulopathy with mortality (Long et al., 2020). There is evidence of both an increase in venous and arterial disease in COVID-19 and many patients have been demonstrated to have antiphospholipid antibodies (Harzallah et al., 2020; Mathian et al., 2020; Reyes Gil et al., 2020; Zhang Y. et al., 2020). 81% of the non-survivors and 76% of survivors received anticoagulation. Our data do not show any differences in outcome based on anticoagulation likely due to size limitations of our small cohort. Indeed, we and others, have demonstrated in a larger cohort that prophylactic anticoagulation reduces mortality (Billett et al., 2020). Despite proper anticoagulation the rate thrombosis is high in COVID inpatients (Al-Ani et al., 2020; Alonso-Fernandez et al., 2020; Demelo-Rodriguez et al., 2020; Helms et al., 2020; Le Jeune et al., 2020; Llitjos et al., 2020). We observed a rate of *in vivo* thrombosis 4.4% (10/225), which is likely an underestimate due to lack of surveillance and limited imaging studies during the COVID peak. Although maximum D-Dimer levels were not significantly different between anticoagulation groups vs. non-anticoagulated patients, maximum D-Dimer levels were significantly higher in patients that developed clots (including *in vivo* and *ex vivo* clots) in both categories discharged and deceased patients. Whereas initial D-Dimer was significantly higher among patients that developed clots and eventually died but not in patients that survived. Interestingly, we observed a higher rate of *ex vivo* thrombosis (mainly in hemodialysis lines) 7% (16/225), significantly higher in non-survivors vs. survivors (16% vs. 2.7%, *p* = 0.0002). This difference is likely a reflection of the higher hemodialysis needs in non-survivors compared to survivors (90% vs. 48%). Nonetheless, the observation of *ex vivo* clots in hemodialysis lines despite anticoagulation suggests that this disease may present with more of a thrombotic microangiopathy (TMA) picture and may be more amenable to TMA therapeutics (Henry et al., 2020; Sweeney et al., 2020; Wang et al., 2020; Cugno et al., 2021). Studies are ongoing looking at therapies with anticoagulation, anti-complement, fibrinolysis (Bikdeli et al., 2020; Laurence et al., 2020).

Modeling showed that an initial D-Dimer value of about 2 μg/ml could distinguish between those that would survive and those that would not. However, sensitivity and specificity of Youden’s cut off were <0.8, indicating that a single initial D-Dimer provides limited information and may need to be coupled with other parameters and/or followed by serial trending. Indeed, analysis of the maximum and minimum levels of important lab parameters indicated that, in addition to the importance of the initial D-Dimer to screen patients that present with coagulopathy and are at higher risk of mortality, the maximum D-Dimer during hospitalization was also associated with mortality. Creel-Bulos et al. (2020) demonstrated that the D-Dimer maximum, magnitude and rate of rise in the first 10 days of admission correlated with VTE but not mortality in a cohort of 115 COVID-19 + inpatients. Similarly, we showed that initial D-Dimer and maximum D-Dimer correlated with clot development but also mortality. The lack of D-Dimer association with mortality in the Creel-Bulos et al. (2020) study may be due to a sample size limitation. Huang et al., showed that an initial D-Dimer = 1 ug/mL correlates with increased risk of mortality. In another cohort from Zhang L. et al. (2020) showed that a D-Dimer cut off = 2 ug/mL better predicted mortality. Similarly, our study showed that a cut off close to 2 ug/mL on initial D-Dimer best stratified our patients at higher risk of mortality. Blacks are known to have higher mean baseline D-Dimers than Europeans and Asians (Naik et al., 2016; Raffield et al., 2017) and the majority of the Hispanics in the Bronx are Afro-Caribbean descendants. Thus, we believe that racial, ethnical, demographic and socioeconomic characteristic are important factors to consider when establishing guidelines utilizing D-Dimer for patient stratification and patient care. Given these data, we would suggest that D-Dimer be factored in the decision-making algorithm of whom to dismiss from the ED. In the Richardson study 2.2% were readmitted within 3 days, although follow-up time was short at 4.5 days (Richardson et al., 2020). Examining the D-Dimer of these patients before discharge may enable us to make more informed decisions. There is evidence that thrombotic and bleeding events may occur in COVID patients post-discharge (Patell et al., 2020); following D-Dimers might be a way to distinguish who should get prolonged thromboprophylaxis. Indeed, the majority of the patients who were readmitted had elevated D-Dimer on readmission (Table 9). 4 of these patients (18% (4/22) among readmitted and 2.7% (4/150) among all discharged patients) had documented thrombosis.

Among the patients that required readmission (22/150, 15%) more than a third died (8/22, 5% of all discharged patients) mainly due to respiratory failure or septic shock. D-Dimer on readmission was available in 4 out of 16 patients that survived readmission and 5 out of 8 patients that died during readmission. In those patients with available D-Dimer that died during readmission, the D-Dimer levels were > 2 ug/ml (Table 6).

Given the strength of D-Dimer as a predictor of mortality, future studies should focus on establishing guidelines on how to use D-Dimer trending in different settings to better predict mortality, monitor disease progression and response to treatment (Hardy et al., 2021).

## Data Availability Statement

The raw data supporting the conclusions of this article will be made available by the authors, without undue reservation.

## Ethics Statement

The studies involving human participants were reviewed and approved by the Albert Einstein College of Medicine IRB committee. Written informed consent for participation was not required for this study in accordance with the national legislation and the institutional requirements.

## Author Contributions

MR and HB conceptualized the study design, performed the methodology, and wrote IRB protocol. JG-L, SR, MB, KI, and JS collected the data and analyzed the data. MR, MB, and JS performed the formal analysis and validation. YL advised on statistical analysis. JS and MB provided visual graphics. MR and HB wrote the manuscript. All authors reviewed, edited, and approved final manuscript submission.

## Conflict of Interest

The authors declare that the research was conducted in the absence of any commercial or financial relationships that could be construed as a potential conflict of interest.
